# *In situ*, Reversible Gating of a Mechanosensitive Ion Channel through Protein-Lipid Interactions

**DOI:** 10.3389/fphys.2016.00409

**Published:** 2016-09-21

**Authors:** Anna Dimitrova, Martin Walko, Maryam Hashemi Shabestari, Pravin Kumar, Martina Huber, Armagan Kocer

**Affiliations:** ^1^Department of Biochemistry, University of GroningenGroningen, Netherlands; ^2^Huygens-Kamerlingh Onnes Laboratory, Department of Physics, Leiden UniversityLeiden, Netherlands; ^3^Neuroscience Department, University of Groningen, University Medical Center GroningenGroningen, Netherlands

**Keywords:** MscL, reversible gating, mechanosensation, electron spin resonance spectroscopy, lysophosphatidylcholines, bovine serum albumin

## Abstract

Understanding the functioning of ion channels, as well as utilizing their properties for biochemical applications requires control over channel activity. Herein we report a reversible control over the functioning of a mechanosensitive ion channel by interfering with its interaction with the lipid bilayer. The mechanosensitive channel of large conductance from *Escherichia coli* is reconstituted into liposomes and activated to its different sub-open states by titrating lysophosphatidylcholine (LPC) into the lipid bilayer. Activated channels are closed back by the removal of LPC out of the membrane by bovine serum albumin (BSA). Electron paramagnetic resonance spectra showed the LPC-dose-dependent gradual opening of the channel pore in the form of incrementally increasing spin label mobility and decreasing spin-spin interaction. A method to reversibly open and close mechanosensitive channels to distinct sub-open conformations during their journey from the closed to the fully open state enables detailed structural studies to follow the conformational changes during channel functioning. The ability of BSA to revert the action of LPC opens new perspectives for the functional studies of other membrane proteins that are known to be activated by LPC.

## Introduction

Elucidating the mechanisms by which ion channels function remains an ongoing challenge in physiology. Even though invaluable structural information is obtained from crystal structures, it is not always possible to derive the conformational transitions necessary for function from the static structures. Therefore, complementary approaches that give information about the dynamic conformers are needed.

**Figure 1:**
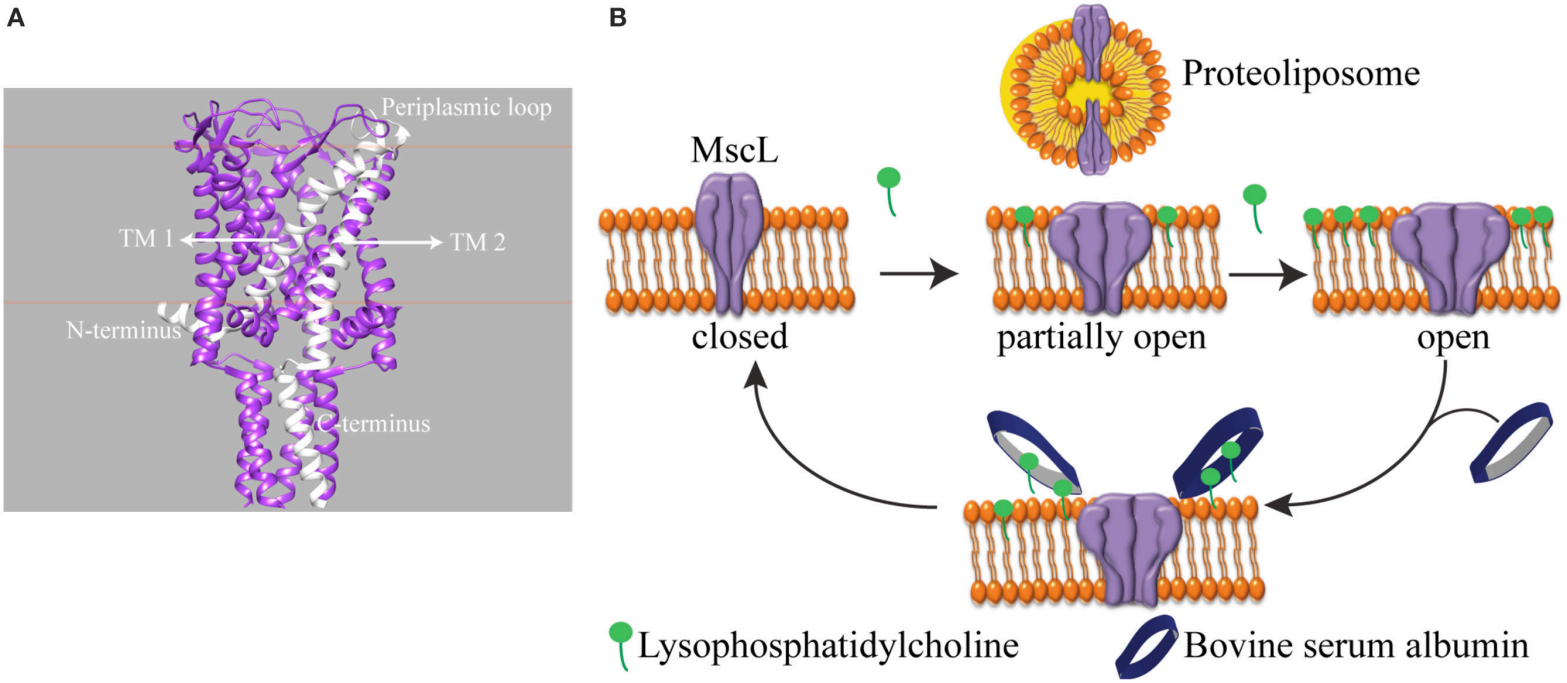
**Schematic representation of reversible activation of MscL**. **(A)** Side view of homopentameric MscL from *Mycobacterium tuberculosis* (PDB access code: 2AOR). The approximate position of the lipid bilayer is marked with orange lines. A single subunit is highlighted in white. (TM: transmembrane domain). **(B)** MscL is reconstituted into the liposome. While the assymmetric insertion of L-α-lysophosphatidylcholine into the lipid bilayer triggers MscL opening, its selective removal by bovine serum albumin (BSA) closes the MscL channel. The open-close cycle can be repeated multiple times. For clarity, only four out of five identical subunits are shown.

We investigated a mechanosensitive ion channel (MSC) from *Escherichia coli* (*E. coli*). In nature, MSC's sense the changes in membrane tension and are involved in hearing, the sense of equilibrium, touch, pain, as well as renal and cardiovascular regulation (Blount et al., 2007). In *E. coli*, mechanosensitive channel of large conductance (MscL) protects cells from a sudden hypoosmotic shock in the external environment by opening a non-selective, temporary pore and releasing small solutes out of the cell (Sukharev et al., 1994). The channel is composed of five identical subunits (Chang et al., 1998; Steinbacher et al., 2007). The N- and C-terminus of the channel is in the cytoplasm, and each subunit has two transmembrane helices connected by a flexible periplasmic loop (Figure 1A). MscL directly translates the mechanical forces in the lipid bilayer into large conformational changes. It does not need any other cell components for its function (Blount and Moe, 1999; Koprowski and Kubalski, 2001). Furthermore, the crystal structures of MscL-homologs in their closed state exist but could not describe the mechanism of channel opening and closing, i.e., gating (Chang et al., 1998; Steinbacher et al., 2007). Recently, we showed that global conformational changes of MscL can be observed using ion mobility native mass spectrometry (Konijnenberg et al., 2014). However, to understand the onset of mechanosensation at the molecular level, there is a need for following the conformational changes from the start of channel opening and closing and relating these conformational changes to the forces in the lipid bilayer. Since the conformational changes leading to full channel opening start even before the channel pore starts conducting ions (Gullingsrud et al., 2001; Gullingsrud and Schulten, 2004), conventional patch clamp electrophysiology, which measures ionic current flowing through an open pore, would miss the initial events. Instead, spectroscopic techniques reporting on the conformational changes would be very useful.

One of the challenges of studying a mechanosensitive ion channel by spectroscopy is to design a tool to control the channel opening and closing. Here we show an approach to achieve this goal. In patch clamp electrophysiology, the membrane can be stretched by applying suction via the recording pipette in a controlled manner. However, this level of control over the channel activity in a spectroscopy setting is missing. To this end, pioneering works by Perozo et al. (2002a,b) explored the lipid bilayer mechanical properties. They showed that reconstituting MscL into lipids with different acyl chain lengths, hence changing the hydrophobic mismatch between the ion channel and the lipid bilayer, lowers the activation energy of MscL and traps the channel in structurally distinct closed intermediate states. On the other hand, asymmetric insertion of an inverted-cone-shaped non-bilayer forming lipid L-α-lysophosphatidylcholine (LPC) into one leaflet of a lipid bilayer (Perozo et al., 2002a,b; Yoshimura and Sokabe, 2010), fixes MscL in its open conformation. In another study, MscL activity was initiated by light in spectroscopic setups (Yilmaz et al., 2015). However, reversible control over channel activity is still missing.

Here, we present a tool that enables reversible activation of MscL *in situ* by LPC and BSA (Figure 1B). In cellular signal transduction, albumins are known to regulate LPC concentration under physiological conditions (Guo et al., 2009) by binding LPC molecules and facilitating their transport. One albumin molecule has a binding capacity of four to five LPC molecules (Thumser et al., 1994; Kim et al., 2007). Indeed, by employing the fluorescence and site-directed spin-label electron paramagnetic resonance (SDSL-EPR) spectroscopy, we show that while the incorporation of defined concentrations of LPC into the liposomes opens MscL into different sub-open states, some of which might not yet be conducting ions, the addition of BSA closes the channels back. Furthermore, based on the relation between the concentration of LPC and tension necessary for activation of MscL (Mukherjee et al., 2014), here we managed to activate MscL to distinct sub-open states by titrating the lipid bilayer with LPC.

We anticipate that reversible control over MscL activity and reaching sub-open conformations by LPC titration will allow to investigate conformational changes of MscL from the onset of channel activation and provide a valuable tool to understand the tension-sensing mechanism of these channels. The findings will pave the way to study other membrane proteins that were shown to be activated by LPC (Marra et al., 2016). Beyond investigating the structure-function relation, technological applications such as biosensors (Kocer et al., 2012; Urban et al., 2014), environment-responsive drug delivery devices (Kocer et al., 2005, 2006; Kocer, 2007; Pacheco-Torres et al., 2015), or membrane-mediated protein-protein communication networks (Charalambous et al., 2012) could also benefit from the reversible control over the activity of such ion channels.

## Materials and methods

### Materials

All common chemicals were of reagent grade and were purchased from Sigma-Aldrich Chemie B. V., Netherlands. (1-oxyl-2,2,5,5-tetramethylpyrroline-3-methyl) methanethiosulfonate (MTSSL) and Methoxypoly(ethyleneglycol)5000 Amidopropionyl Methanethio-sulfonate (MTS-PEG5000) were purchased from Toronto Research Chemicals Inc. (TRC), Toronto, Canada. Azolectin was from Avanti Polar Lipids. Polycarbonate membrane was from Avestin Europe, GmbH. Biobeads (SM-2 Absorbents) were from Bio-Rad Laboratories, Inc., CA, USA.

### Site-directed spin labeling

G22C mutant of MscL channels was produced and isolated as described before (Kocer et al., 2007). MTSSL was used as the spin-label. The purified MscL channels were labeled with MTSSL either maximally or minimally with MTSSL to protein (mol/mol) ratio of 5:1 or 1:5 (mol/mol) as explained before (Yilmaz et al., 2015). Briefly, labeling was performed by incubating the necessary amount of the spin label and detergent-solubilised MscL at room temperature for 30 min with gentle rotation. The labeling was stopped by removing the unbound MTSSL by Sephadex G-25 size exclusion column chromatography.

### Spin labeling efficiency

The spin labeling efficiency (SLE) was calculated as explained before (Yilmaz et al., 2015). Briefly, the labeling was confirmed by the electrophoretic mobility shift assay (EMSA) and quantified by EPR. For the EMSA, the spin-labeled MscL (MscL-SL) was incubated for 5 min with β-mercaptoethanol-free SDS-PAGE sample buffer. This step dissociates MscL into its monomers and thereby exposes the free cysteines. Next, a cysteine-specific high molecular weight compound MTS-PEG5000 was added to the sample to a 2 mM final concentration. Subsequently, the protein was separated on a 12.5% SDS/PAGE gel. For quantification, the gel was scanned using a Fujifilm LAS-3000 imager and was analyzed by using AIDA image analyzing software (Raytest GmbH).

The SLE was quantified by comparing the integral of the EPR absorption spectra of the labeled protein and a reference spin probe MTSSL of known concentration by using Equation (1):

(1) $$C_{samp}=\frac{N_{samp\;\cdot }C_{ref}}{N_{ref}}$$

Where, ***C***_*samp*_ is the concentration of MTSSL in the labeled protein, ***N***_*samp*_ and ***N***_*ref*_ are the evaluated integrals of the absorption spectra of the sample and the reference, respectively, and ***C***_*ref*_ is the reference concentration. Typically, 100 mM MTSSL dissolved in DMSO was used as a reference. Then, *C*_*samp*_ was used to calculate the SLE by Equation (2):

(2) $$SLE=\frac{C_{samp}}{C_{prot}}$$

Where, *C*_*prot*_ is the concentration of the labeled protein determined by the standard Bradford assay.

### Reconstitution of MTSSL-Labeled G22C MscL into liposomes

The protein was reconstituted into liposomes as previously described (Kocer et al., 2007), with slight modifications. Briefly, 20 mg/ml azolectin in a lipid buffer (150 mM NaCl, 10 mM sodium phosphate buffer, pH 8.0) was subjected to five freeze-thaw cycles in liquid nitrogen and 50°C, respectively. The liposomes were sized by extrusion through a 400 nm polycarbonate membrane. The resulting large unilamellar liposomes were saturated by the addition of final 9 % (v/v) detergent (Triton X-100). Labeled protein and detergent-saturated liposomes were mixed at 1:50 molar ratio and incubated for 30 min at 50°C. Subsequently, either 10 mM sodium phosphate buffer, pH 8.0 (for EPR measurements) or 200 mM calcein (for fluorescence measurements) was added in 1:1 volume ratio and supplemented with 6 mg (wet weight) Biobeads (SM-2 Absorbents) per 1 μL detergent (10% Triton X-100). For detergent removal, the sample was incubated overnight at 4°C under mild agitation.

For EPR experiments, proteoliposomes were concentrated by centrifugation at 135,000 g for 50 min at 4°C and resuspended in 20–50 μL of 10 mM sodium phosphate buffer, pH 8.0.

### Fluorescence dequenching assay

Before the fluorescence dequenching experiments, the reconstituted calcein-loaded proteoliposomes were subjected to a size exclusion chromatography using Sephadex G50 matrix for separating the external calcein. The matrix was equilibrated with the efflux buffer (10 mM sodium phosphate buffer, pH = 8; 150 mM NaCl; 1 mM EDTA). Next, a 2 μL of proteoliposomes were added to a cuvette filled with 2100 μL efflux buffer. Fluorescence emission was monitored at 515 ± 2 nm (excitation at 495 ± 2 nm) in a Cary Eclipse Fluorescence Spectrophotometer (Varian Inc.). Channel activation at different LPC concentrations was achieved by the addition of LPC to the sample and followed for approximately 3 min. Channel deactivation was achieved by adding BSA to already LPC-containing sample and followed for 1 min. Six different final LPC concentrations were tested: 1, 1.5, 2.0, 2.5, 3.0, and 3.5 μM, whereas 25 mol% BSA with respect to LPC concentration was added for channel deactivation. At the end of each experiment, proteoliposomes were lysed by the addition of Triton X100, and the resulting fluorescence value was taken as 100 %.

The release of calcein was calculated as a percentage of the total calcein amount present in the proteoliposomes:

(3) $$\%\text{ Release }=\;{\left[(\text{I}-{\text{I}}_{\text{o}})/({\text{I}}_{100}-{\text{I}}_{0})\right]}^{*}100$$

Where, I is the measured fluorescence intensity at a given time, Io is the initial background fluorescence intensity, and I_100_ is the fluorescence intensity upon complete lysis of the proteoliposomes, which was elicited by adding 100 μL Triton X-100 from a 10% stock.

### EPR spectroscopy

Continuous wave (cw) EPR measurements were performed using a MiniScope benchtop X-band EPR spectrometer (MS400 Magnettech GmbH, Berlin, Germany) with a rectangular TE102 resonator. The cavity was fluxed with gaseous nitrogen to keep the temperature stable. The microwave power and the B-field modulation amplitude were set to 10 mW and 0.20 mT, respectively (see supplementary information for determination of overmodulation in this particular instrument). EPR glass capillaries with 0.9 mm inner diameter were filled with a sample volume of 10 μL (for LPC) and 14 μL (for BSA) at a final MscL concentration of 170–200 μM.

The microwave frequency was 9.41 GHz; the modulation frequency was 100 kHz. Each spectrum of maximally labeled protein corresponds to the accumulation of 16 scans while spectra of minimally labeled protein correspond to the accumulation of 25 scans.

### Channel activation and deactivation followed by EPR

EPR experiments at increasing LPC concentrations were carried out from a single stock of spin labeled MscL (MscL-SL) reconstituted in liposomes. The stock was divided into six aliquots (including one control sample, i.e., labeled and reconstituted MscL-SL without LPC addition). A desired LPC to azolectin ratio was obtained by adding the required amount of LPC to a constant final volume (10 μL) always 1 h before starting the EPR experiment. Channel closure was achieved by adding BSA to LPC-activated channels at 25 mol% with respect to LPC concentration (LPC: BSA molar ratio) to 14 μL final volume.

### Analysis of EPR spectra

To determine spin-label mobility the inverse peak-to-peak central linewidth ΔH^−1^ and the second moment <ΔB^2^> were used. After base-line correction, using the MS400–Analysis software (Magnettech GmbH, Berlin, Germany), the first and second moments of the EPR spectra were calculated numerically using the UNISPC program (provided by Dr. Johann Klare, Universität Osnabrück, Germany).

The difference in spectral second moment Δ <ΔB^2^> was calculated according to the following equation (Steinhoff, 2002):

(4) $$\begin{array}{l} \Delta <\Delta {\text{B}}^{2}>=<\Delta {\text{B}}_{I}{}^{2}>-<\Delta {\text{B}}_{N}{}^{2}>\\ \;=\frac{\int {\left(B-B_{FI}\right)}^{2}S_{I}(B)dB}{\int S_{I}(B)dB}-\frac{\int {\left(B-B_{FN}\right)}^{2}S_{N}(B)\mathrm{dB}}{\int S_{N}(B)\mathrm{dB}} \end{array}$$

Where < ΔB_*I*_^2^ > and < ΔB_*N*_^2^ > are second moments of dipolar-broadened (interacting spins) and non-broadened (non-interacting spins) EPR spectra, respectively; *S*_*I*_(*B*) is the EPR absorption spectrum in which the dipolar spin-spin interaction is present, whereas *S*_*N*_(*B*) is the spectrum without spin-spin interaction; *B*_*FI*_ and *B*_*FN*_ are the first spectral moments of the respective spectra, and *B* is the static magnetic field.

## Results

### MscL can be reversibly activated in liposomes

**Figure 2:**
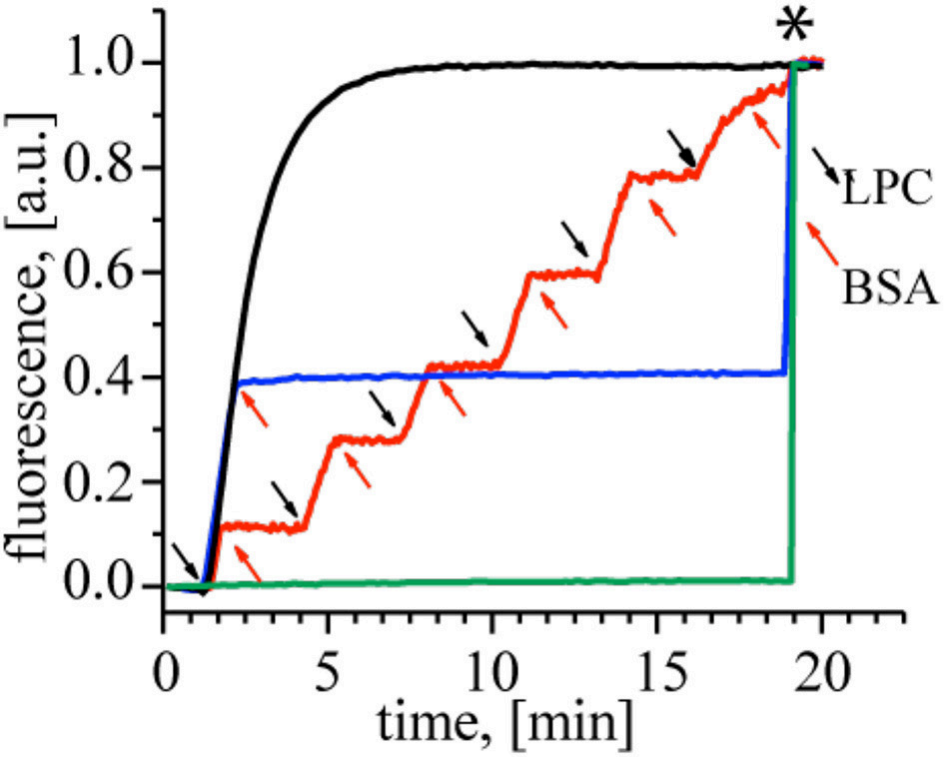
**Reversible activation of MscL by LPC and BSA**. Black trace: Calcein loaded control MscL proteoliposomes treated with 40 mol% LPC reaches maximum calcein release within 5 min. When the duplicate of the control sample was treated with 25 mol% BSA (relative to LPC concentration) at *t* = 2 min, the calcein release stopped immediately (*blue trace*). The *red trace* shows repeated opening and closing cycles of MscL. Consecutive LPC and BSA additions indicated by black and red arrows, respectively. Calcein-loaded liposomes with no MscL channel were used as a negative control to test the effect of 40 mol% LPC (relative to the lipid concentration) and 25 mol% BSA (relative to LPC) on the lipid bilayer itself (*green trace*), showing that the indicated amounts of LPC and BSA did not cause any leakage through the lipid bilayer. The asterisk marks the time point of detergent addition to the samples for lysing the liposomes and releasing all calcein.

First, to demonstrate the action of LPC and BSA on MscL, we performed a fluorescence dequenching assay using MscL reconstituted into liposomes (Kocer et al., 2007). Insertion of LPC into the outer bilayer leaflet was achieved by adding a defined concentration of LPC to freshly prepared G22C MscL proteoliposomes that were loaded with a self-quenching fluorescent dye, calcein. The dye is released through MscL channels and the concomitant increase in fluorescence is followed in time. Figure 2 shows that upon the addition of 40 mol% LPC (relative to lipid concentration), the calcein release reaches a plateau within 5 min (*black line*). If, on the other hand, BSA (25 mol% with respect to LPC concentration) was added to a duplicate of the first sample before the release reaches the plateau, the release stopped immediately (Figure 2, *blue line*), indicating that LPC and BSA can be used to open and close the channel, respectively. We showed that the cycle of opening and closing of the channel could be repeated multiple times, as shown with the *red line* in Figure 2. Together, the results show a lipid–mediated reversible control of MscL gating.

### MscL requires less than 10 mol% LPC for its activation

**Figure 3:**
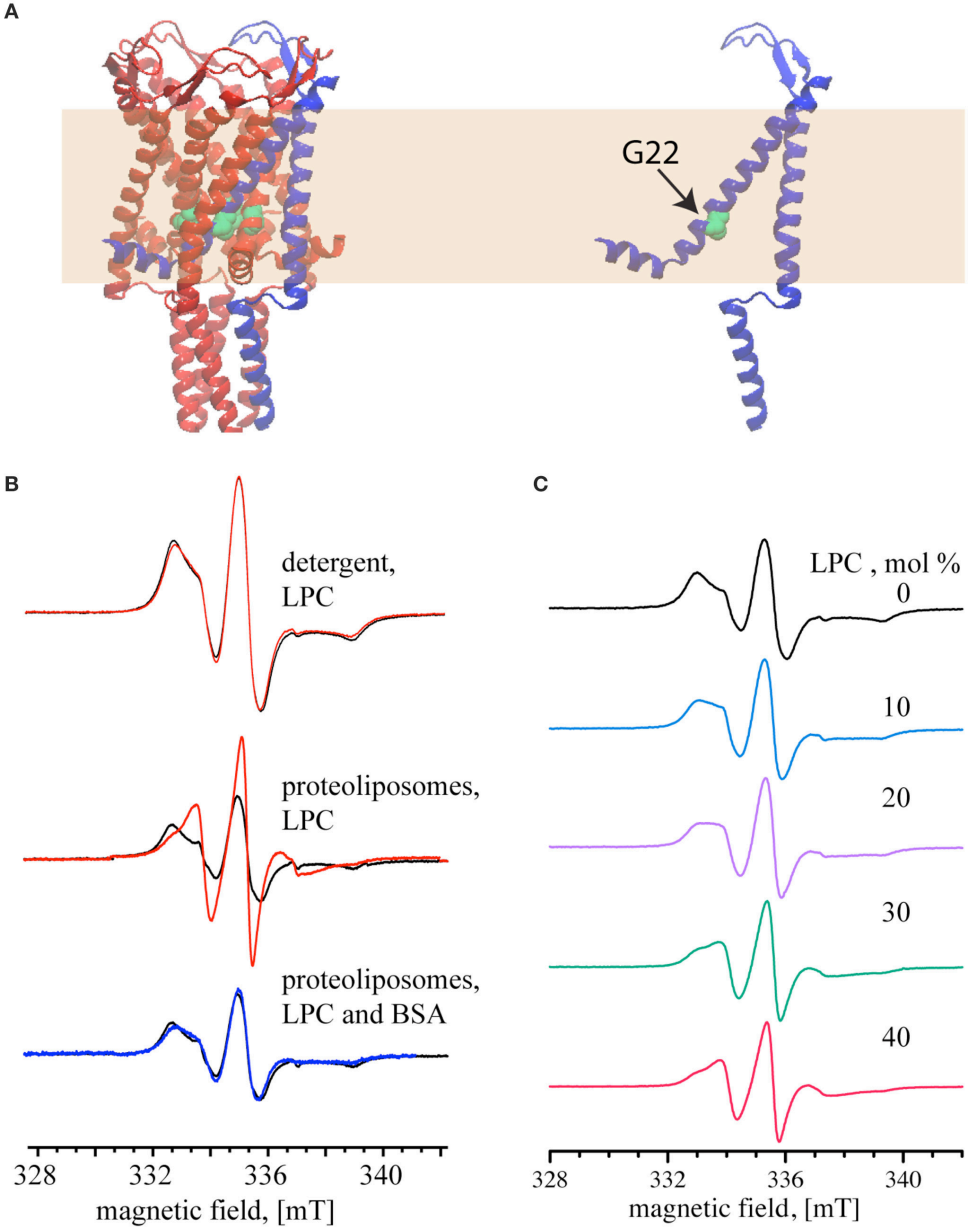
**Reversible activation of MTSSL-labeled G22C MscL monitored by continuous wave EPR**. **(A)** Schematic representation of MscL based on the crystal structure of the homologous MscL from *Mycobacterium tuberculosis* (PDB access code: 2AOR). The yellow area represents the lipid bilayer. **(B)** MscL channel activation by 40 mol% LPC (relative to the lipid concentration). *Top and Middle*: the spectra show MTSSL-labeled G22C MscL (MscL-SL, minimally labeled) in the absence of LPC (*black*); activated with LPC (*red*). *Bottom*: the spectra of LPC-activated channels after adding BSA to the proteoliposomes (*blue*). Spectra are normalized to the same number of spins. **(C)** EPR spectra of maximally labeled MscL-SL samples with increasing mol% of LPC, percentages are given relative to the lipid concentration. The magnetic field scale shown at the bottom of **(B,C)** refers to all spectra of the respective set.

Next, we tested reversible activation of MscL by employing EPR spectroscopy. In our experiments, we monitored the mobility of a pore amino acid of MscL upon activation and deactivation by LPC-BSA. To perform the EPR experiments, the cysteine at the 22nd position of MscL (see Figure 3A) was labeled either maximally or minimally with ((1–oxyl–2,2,5,5–tetramethylpyrrolin–3–methyl) methanethiosulfonate) (MTSSL) (Altenbach et al., 1989). The choice of the G22 position is based on its critical location in the pore (Yoshimura et al., 1999), its high accessibility for labeling (Kocer et al., 2005, 2006), and the significant increase in spin-label mobility at this site upon channel activation (Perozo et al., 2002a,b). The MTSSL labeling of MscL was confirmed by an EMSA (see Supplementary Figure 1) and quantified by employing EPR spectroscopy. The SLE for the maximally and minimally labeled samples were 88 and 18 %, respectively.

After showing that LPC has no effect on detergent solubilized MscL (Figure 3B, *top panel*), the channels were reconstituted into liposomes and used for EPR measurements. The *black line* in the *middle* and *bottom panel* of Figure 3B shows the spectra of the maximally labeled channel in its initial closed state. Addition of 40 mol% LPC relative to the lipid concentration (opening of the channel) significantly narrowed the line width of the EPR spectrum (Figure 3B, *middle panel, red line*). Increase of the LPC concentration above 40 mol% did not change the EPR spectrum anymore. The addition of 25 mol% BSA (relative to LPC concentration), on the other hand, generated a signal that completely reverses the changes seen in the middle panel and results in a spectrum that is very similar to that of the closed MscL channel (Figure 3B, *lower panel, blue line*).

After showing that 40 mol% LPC opens the channel fully, and that the EPR spectra differ significantly for the closed and the open channel, we set out to determine, if intermediate opening steps of the MscL channel can be reached by adding LPC at concentrations below 40 %. Indeed, EPR line shape changes can be observed at concentrations as low as 10 mol%. Between 10 and 30 mol% LPC the EPR line shape changes continuously, and the 30 mol% spectrum is identical to the one at 40 mol% LPC (Figure 3C).

### Channel opening monitored by EPR

**Figure 4:**
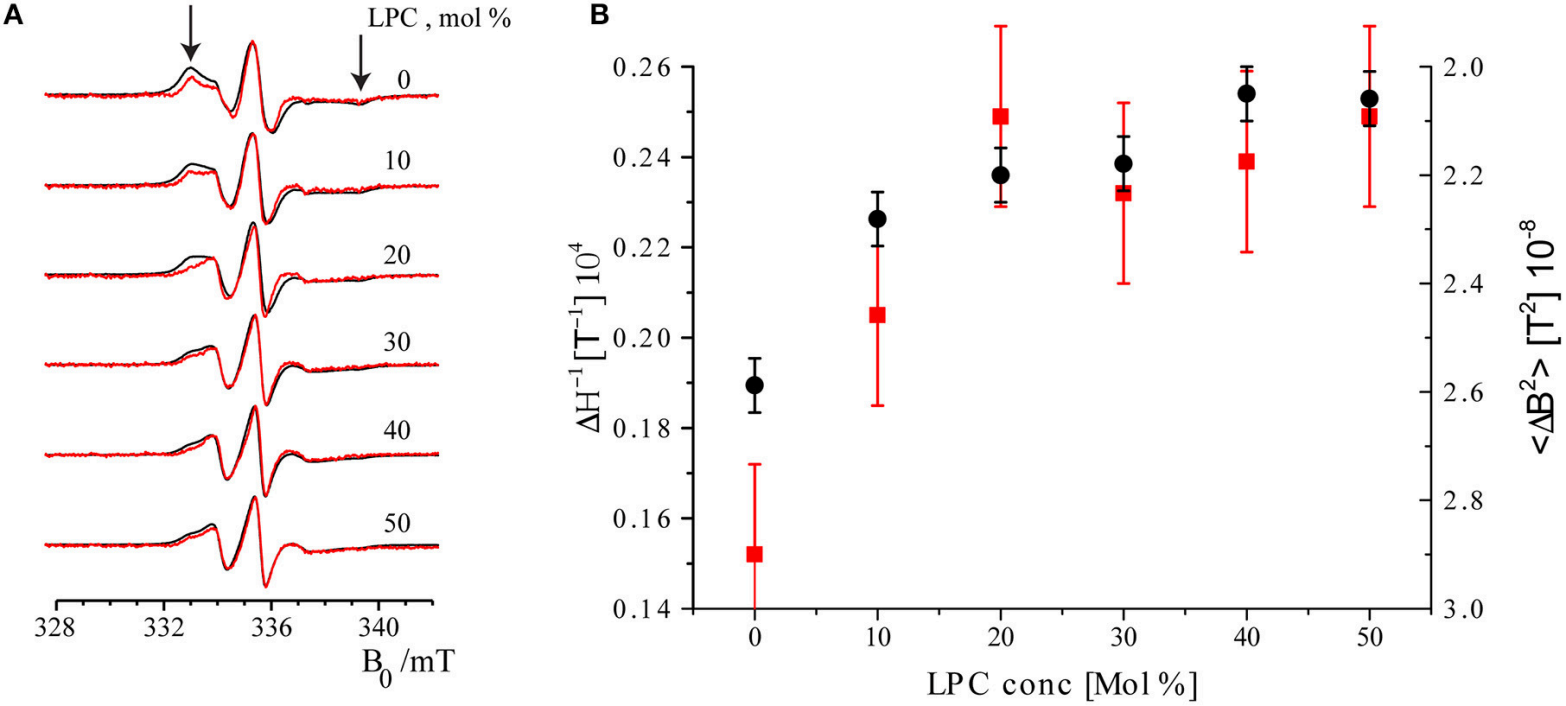
**Room temperature cw EPR spectra of minimally labeled and maximally labeled MscL-SL**. **(A)** *Black*: maximally labeled, *red*: minimally labeled MscL. LPC concentrations in the liposomes are indicated. Spectra are normalized to the amplitude of the central line. The arrows indicate features of the spectra that derive from spin labels that are slowly rotating (see text). The features indicated by arrows diminish and disappear upon increasing the concentration of LPC. **(B)** Parameters describing spin label mobility as a function of LPC concentrations: Inverse central line width (ΔH^−1^, red squares) and second moment (<ΔB^2^>, black dots) of the minimally labeled sample. Increased mobility is reflected in a narrower line, which results in a larger inverse central line width (ΔH^−1^), and a smaller second moment (<ΔB^2^>) parameter.

The EPR spectra of maximally and minimally labeled MscL samples (Figure 4A, *black and red, respectively*) revealed a narrowing of the spectral lines with increasing LPC concentration. In addition, the hallmarks of the slow rotation in nitroxide EPR spectra, as marked by *arrows* in Figure 4A on the high and low field side of the spectra, diminished and finally disappeared. Between 20 and 30 mol% of LPC, especially the spectra of the maximally labeled sample change, whereas those of the minimally labeled one are almost identical.

To quantitate the changes in EPR line-shape, two mobility parameters, inverse central line width (ΔH^−1^) and second moment (<ΔB^2^>), were calculated from the EPR spectra of the minimally labeled sample. The inverse central line width increases as the mobility of the spin label increases, since less mobility causes broadening of the EPR lines. The second moment (Slichter, 1992) is a measure of the overall width of the EPR spectrum that can be determined for a series of spectra also in the presence of overall line shape changes, so it is a robust measure of the broadening. Also common in the literature is to use the inverse second moment as a measure of mobility, as introduced by the Hubbell group (Mchaourab et al., 1996). In Figure 4B these parameters are plotted as a function of LPC concentration. Both parameters show the increase in spin-label mobility up to LPC concentrations of 30 mol% and then remain constant within the experimental error (see Figure 4B). Together, these results show that the spin-label mobility increases as the LPC concentration increases.

The second trend manifests itself in a broadening of the EPR spectra of the maximally labeled (Figure 4A, *black*) with respect to the minimally labeled (Figure 4A, *red*) EPR spectra LPC concentrations up to 30% of LPC. This is most obvious in the spectrum with 20 mol% LPC at field values around 333 mT (Figure 4A, *the left arrow*).

Line broadening in the maximally labeled with respect to the minimally labeled sample suggests that there is a spin-spin interaction in the maximally labeled sample when the channel is in its closed state (i.e., at low LPC concentrations, i.e., ≤10 %). However, as the LPC concentration increases, the spin-spin interaction between spin labels attached to different subunits of MscL reduces, suggesting pore opening. A measure for the amount of broadening of the EPR spectra is the difference in spectral second moments (Equation 4), Δ <ΔB^2^>, the values of which are given in Table 1.

**Table 1:** **Effect of spin-spin interaction on the EPR line shape of MscL-SL**.

**LPC conc**. **[mol%]**	**Δ <ΔB^2^> × 10^−10^** **[T/^2^] ± 20 × 10^−10^**
0^*^	99
10	146
20	145
30	84
40	98
50	82

Line broadening is expressed as the second moment difference Δ <ΔB^2^> in T^2^ obtained from spectra of maximally and minimally labeled MscL.

* The second moment of the maximally labeled sample is smaller than expected considering the values at 10 and 20% LPC, possibly due to a not fully closed channel in this particular sample, resulting in a too small Δ <ΔB^2^> value.

Although overall small, the Δ <ΔB^2^> values show a sharp decrease between 20 and 30% LPC concentration suggestive of channel opening (for details, see Discussion). The difference in second moment values at and above 30 mol% LPC are so small, that spin-spin interaction is considered to be negligible.

## Discussion

In the present study, we could reversibly control MscL activity by exploring the LPC–BSA interplay on the lipid bilayer. The only other method allowing reversible control on this channel *in situ* was a reversible light switch, which was covalently linked to the pore region of MscL (Kocer et al., 2005). However, spectroscopy probes, such as MTSSL, also attach to the protein via cysteine chemistry, which complicates the channel labeling (Yilmaz et al., 2015).

With the present approach, we showed the feasibility of titrating MscL channel opening to different sub-open states by dose-dependent activation of the channel by LPC. Previously, high concentrations of LPC has been used to open MscL completely to study the conformational changes of the pore forming TM1 helix (Perozo et al., 2002a,b) and, recently, the N-terminus of MscL (Bavi et al., 2016). Interestingly, despite its dramatic effect on the channel opening, the mechanism of LPC-induced channel activation is still debated (Yoshimura and Sokabe, 2010). The partitioning of LPC changes the lipid bilayer properties (Zhelev, 1998) some of which have been proposed as the mechanism of LPC effect on MscL. such as changes in the lateral pressure profile (Perozo et al., 2002a; Esteban-Martín et al., 2009), bending of the membrane (Wiggins and Phillips, 2004), and changes in the surface tension (Yoshimura and Sokabe, 2010). In a recent work, we have shown that LPC does not interact with MscL to exert its effect but requires the lipid bilayer. However, its action cannot solely be explained by the changes in the lipid bilayer properties (Mukherjee et al., 2014), either. While the amount of LPC partitioning in a liposome would mimic the magnitude of applied tension in the patch clamp by discriminating tension-sensitive mutants of MscL from *E. coli*, it failed in distinguishing MscL from *E. coli, Mycobacterium tuberculosis*, and *Lactococcus lactis*, based on their tension-sensitivity. Therefore, it has been suggested that LPC exerts its effect on the MscL by changing the protein-membrane coupling, as also proposed by others (Lee, 2004; Marsh, 2008; Lundbaek et al., 2010).

To ensure maximum sensitivity of the EPR spectra to the changes occurring at different sub-open states, we designed the study to be sensitive to two properties: the spin label mobility and the spin-spin interaction, similar to approaches presented earlier (Der-Sarkissian et al., 2003; Margittai and Langen, 2006; Scarpelli et al., 2009). The mobility of the spin label at channel position 22 increases stepwise and in a dose dependent way, with increasing concentrations of LPC, showing stepwise opening of the channel (see Figures 1, 3A, 4B). As the channel opens, its pore widens, providing more space for the spin label to move. The observed mobility increase is in agreement with previous EPR studies on the fully open channel (Perozo et al., 2002a,b).

Similarly, the spin-spin interaction, which depends on the distance between the spin labels, decreases, showing an increase in the distance between the spin labels as the LPC concentration increases. Again this decrease in spin-spin interaction is expected as the channel opens, since the widening of the pore increases the distance between the protein subunits to which the spin labels are attached. Quantification of the increase in distance (Steinhoff, 2002) between the subunits, and thereby structural information of the channel in different open states, cannot be obtained under the conditions of the present study, because of the labeling conditions employed. In the Supplementary Material we give a detailed account of why distances cannot be extracted and describe how specific assembly of channels, as described in Birkner et al. (2012) would solve this issue.

Together with the ability to open the channel to intermediate states, our approach allows to close the channel from any open state back to the closed conformation. This will enable detailed structural studies in the future and will provide a more complete picture of the gating mechanism of MscL from the onset of the mechanosensation.

## Author contributions

MW, AD, and AK designed the research, MW, AD performed research, PK and MHS performed experiments and provided analysis methodology, MW, AD, MH, AK analyzed data, and all authors contributed to writing the article.

## Funding

Financial support by the Foundation for Fundamental Research on Matter (FOM), which is part of the Netherlands Organisation for Scientific Research (NWO) (to MH) and by European Research Council (Starting Grant 208814 to AK) is gratefully acknowledged.

### Conflict of interest statement

The authors declare that the research was conducted in the absence of any commercial or financial relationships that could be construed as a potential conflict of interest.
