# Supplementary material for: In situ, Reversible Gating of a Mechanosensitive Ion Channel through Protein-Lipid Interactions
Source: Front Physiol. 2016 Sep 21;7:409. doi: 10.3389/fphys.2016.00409 (PMC5030285; doi:10.3389/fphys.2016.00409)
Supplement: Supplementary file 1 [file DataSheet1.PDF]

Supplementary Material

In situ, Reversible Gating of a Mechanosensitive Ion Channel through Protein-Lipid Interactions

Anna Dimitrova, [†] **Martin Walko,**[†] **Maryam Hashemi Shabestari**[‡]**, Pravin Kumar**[‡]**, Martina Huber,**[‡*] **and Armagan Kocer***

Correspondence: a.kocer@umcg.nl, huber@physics.leidenuniv.nl

1 Electrophoretic mobility shift assay

The spin-labeled MscL (MscL-SL) was incubated for 5 min with β mercaptoethanol-free SDS-PAGE sample buffer. A cysteine-specific high molecular weight compound MTS-PEG5000 was added to the sample to a 2 mM final concentration. Subsequently, the protein was separated on a 12.5% SDS/PAGE gel. If the cysteine (G22C) of a particular MscL subunit has already an attached MTSSL spin label to it, MTS-PEG5000 cannot bind to that cysteine. Hence, the molecular weight of the subunit stays unchanged. However, if the cysteine was not occupied, the binding of MTS-PEG5000 increases the molecular weight of the monomer.

The fully spin-labeled MscL (Fig. S1, *lane 1*) had no free cysteines to interact with MTS-PEG5000 and therefore, it stayed at the expected monomer molecular weight after incubated with MTS-PEG5000 (Fig. S1, *lane 2*). However, the underlabeled MscL (Fig. S1, *lane 3*) had number of unoccupied cysteines, which could interact with MTS-PEG5000. The underlabeled MscL, therefore, gave two populations of monomers, the one with the expected molecular weight and the other with a higher molecular weight due to the added PEG5000 (Fig. S1, *lane 4*) (2). The absence of a changed molecular weight fraction for sample labeled with a fivefold excess of MTSSL, and the presence of such a fraction for the sample labeled with a 1 to 5 ratio of MTSSL to protein, shows that the former was fully MTSSL-labeled, whereas the latter contained free cysteines, i.e. was underlabeled.

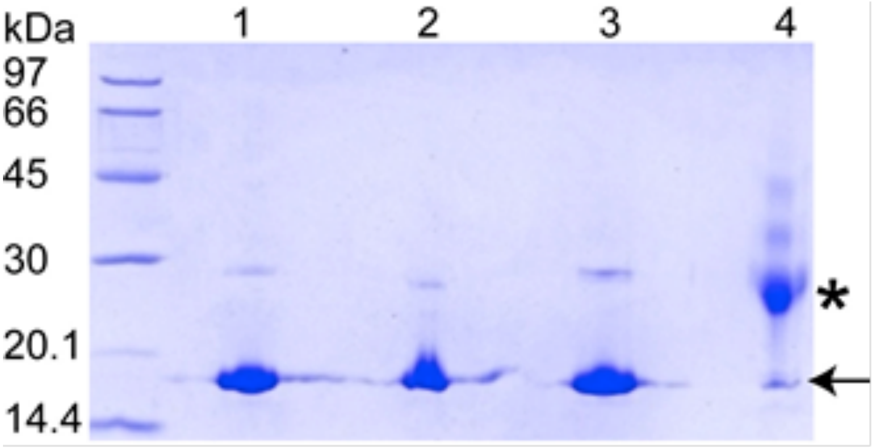

Supplementary Figure 1. Coomassie blue stained 12,5% SDS-PAGE gel. *Lane 1* – maximum labeled MscL with MTSSL; *Lane 2* – sample from lane 1 incubated with MTS-PEG5000; *Lane 3* – underlabeled MscL with MTSSL (1:5 mol/mol MTSSL:

MscL monomer); *Lane 4* - sample from lane 3 incubated with MTS-PEG5000; The final concentration of MTS-PEG5000 in all cases is 2 mM. The *arrow* indicates expected subunit molecular weight of MscL, *star* indicates shifted molecular weight of an MscL monomer that is carrying a PEG-5000 moiety.

2 Analysis of spin-spin interaction

The broadening of the EPR spectra of the maximally labelled compared to the minimally labelled sample at LPC concentrations below 30 mol% shows that spin-spin interaction is detectable in the closed and slightly open state of the channel.

This broadening suggests that the spins in multiply spin labelled channels have a shorter distance with respect to each other in the closed compared to the open channel. In principle, distance information could be obtained from the amount of broadening by equation S1, which relates the interaction of a pair of electron spins, $\Delta<\Delta B^2>$, to the distance as (Steinhoff, 2002)

$$\Delta < \Delta B^2 >= p\frac{1}{r^6} \qquad (S1)$$

where r is the distance between the two unpaired electron spins and p is a constant ($\approx 1.56\text{x}10^{-60}\ T^2m^2$).

Application of this equation requires that the second moment of the reference sample is devoid of dipolar interaction. We show in the following that this is not the case for the minimally labelled sample of MscL.

Labelling degree of minimally labeled sample.

The distribution of **p** labels over **n** protein subunits is given by

$$\frac{n!}{(n-p)!\,p!}\cdot a^p \cdot (1-a)^{n-p} \qquad (S2)$$

with **a** the labelling degree. For the pentamer (**n** = 5), at a labelling degree of **a** = 18 %, assuming uniformly random labelling, 41 % singly labelled, 18 % doubly, and 3 % triply labelled pentamers are expected, showing that only 41 % of the spin labels are non-interacting, since this is the fraction of singly labelled pentamers. Neglecting triply labelled channels 36 % of the spin labels observed in the minimally labelled sample are interacting (two spin labels for each doubly labelled MscL). In the closed state of the channel (for distances see below) this will lead to significant dipolar interaction, making it a poor reference for the second moment of the non-interacting spin-label.

Consequences of labelling degree

By the same reasoning, the maximally labelled sample is not uniformly five-fold labelled, which decreases the $<\Delta B^2>$ values of the maximally labelled samples from those expected for five interacting spins, which, together with the larger second moment for the minimally labelled sample explains that the values of $\Delta<\Delta B^2>$ are smaller than what would be expected and show little spread upon channel opening.

It is therefore not surprising that the application of eq. (S1) to the largest $\Delta<\Delta B^2>$ parameter measured (at 10 % LPC concentration, Table 1, main text), results in a

distance of 2 nm. This distance, which only accounts for one spin pair and not even a pentamer, is much longer than the distance of 8.24 Å between the Cα atoms of residue G22 in two direct neighbors in the pentamer, or 13.33 Å, the distance between second nearest neighbors in the X-ray structure of the closed channel (PDB access code: **2AOR**), illustrating that the $\Delta<\Delta B^2>$ values in Table 1 reflect only a minor fraction of the spin-spin interaction**.** By virtue of this, also the $\Delta<\Delta B^2>$ values are so small, that any further analysis, for example including the multiple-spin interaction that would describe the $\Delta<\Delta B^2>$ properly, is not useful.
These results show, however, that even cw-EPR would be sufficient to extract distances, if the samples were prepared by directed assembly of singly, respectively maximally labeled constructs by biochemical means (Yilmaz et al., 2015) .

3 Testing for possible overmodulation ofMiniscope 400

The overmodulation of the bench-top EPR spectrometer Miniscope 400 was determined as follows. 10µM TEMPOL, a nitroxide radical, in DMSO was measured at different modulation amplitudes (B***m***) in the range of 0.05mT to 0.5mT. The relation between B***m,*** the line width and the signal intensity was followed. The natural line width of TEMPOL is 0.17 mT. As can be seen in the Table below, in Miniscope 400, when the B***m*** is set 88% (i.e. 0.15mT) from the natrural line-width, the signal is not overmodulated and the intensity increases by factor of 2.4. In our experiment, we first 'tested' the EPR line-width with Bm=0.15mT (at Bm = 0.10mT, the signal of the minimally-labelled protein was very low with a signal-to-noise ratio of ~ 5-6 after 36 accumulations). Comparison between the EPR spectra at B***m*** = 0.15mT and 0.2mT showed a difference in the signal intensity, while no difference in the EPR line shape.

The narrowest line-width in this study was 0.46mT, i.e, the used B***m*** was $B_m \leq 50\%_\Delta B_{pp}$ (where, B_m is the modulation amplitude, and $_\Delta B_{pp}$ is the peak-to-peak first-derivative line width), which gave a high signal intensity without affecting the natural line with and any worry of overmodulation.

B_m [mT]	line width [mT]	relative intensity [a. u.]
0.050	0.1706	18.22
0.100	0.1663	33.47
0.150	0.1807	43.79
0.175	0.1981	47.94
0.200	0.2053	50.96
0.250	0.2299	52.55
0.300	0.2675	53.97
0.350	0.3123	53.21
0.400	0.3123	53.21
0.500	0.4540	50.20

References

Steinhoff, H.-J. (2002). Methods for study of protein dynamics and protein-protein interaction in protein-ubiquitination by electron paramagnetic resonance spectroscopy. *Front. Biosci.* 7, c97–110.

Yilmaz, D., Dimitrova, A. I., Walko, M., and Kocer, A. (2015). Study of light-induced MscL gating by EPR spectroscopy. *Eur Biophys J*, 1–11. doi:10.1007/s00249-015-1063-4.
